# Hydro-morphological behavior around T-shaped spur dikes with downward seepage

**DOI:** 10.1038/s41598-023-37694-w

**Published:** 2023-06-28

**Authors:** Harish Kumar Patel, Bimlesh Kumar

**Affiliations:** Department of Civil Engineering, Indian Institute of Technology Guwahati (IITG), Guwahati, India

**Keywords:** Civil engineering, Limnology

## Abstract

The present work experimentally analyses the flow behaviour near the T-shaped spur dike field with no seepage, 5%, and 10% downward seepage. Experiments were aimed at analysing the channel morphology with different discharges. According to the results, downward seepage movement causes significant modification in the channels bed elevation and the development of scour depth. The maximum scour depth is observed at the edge of the first spur dike facing the flow. The rate of scouring also increases with the effect of seepage. Due to downward seepage, the flow distribution is shifted near the channel bed. However, near the channel boundary attained some velocity, significantly enhancing the sediment transport rate. The wake zone between the spur dikes saw very low-velocity magnitudes of positive and negative values. This reveals secondary current generation inside the loop and cross-stream circulation. With an increment of seepage percentage, the velocity, Reynold shear stress, and turbulent kinetic energy magnitude also rise close to the channel’s boundary.

## Introduction

Riverbank erosion is a natural process that occurs when water in a river causes the soil along the banks to erode and be carried away downstream. Riverbank erosion is a common problem that can lead to the instability of riverbanks and even the collapse of adjacent structures like bridges, buildings, and roads^1^. The consequences of riverbank erosion can be severe, not only for the environment but also for human populations living near rivers. Erosion can lead to the loss of agricultural land, damage to infrastructure, and destruction of wildlife habitats^2–5^. Moreover, the sediment carried away by the river during erosion can cause downstream problems such as increased sedimentation, reduced water quality, and increased flooding risks. Therefore, it is essential to understand the mechanism behind riverbank erosion and to develop effective measures to control and regulate it. Many researchers’ findings focus on understanding the mechanism behind the river bank erosion and flow characteristics near the bank^6–10^. At the same time, some research focuses on quantifying the annual erosion rate of riverbanks^11, 12^.

River training works, which involve the construction of various structures on a river to guide and confine the flow, are one way to provide stability for flow characteristics and reduce the risk of bank erosion. Different structures, such as spurs or groynes, levees, and guide banks, can be built to provide stability to the channel’s cross-section and to safeguard the bank from erosion. Among these structures, the spur dike offers the best solution for protecting the bank^13, 14^. It is projected outward from the river bank to prevent erosion by controlling and diverting the flow from the river bank. They are designed based on project requirements and engineering considerations. The process involves site assessment, design, construction, and monitoring. The engineer’s experience is important in selecting the right design due to variations in hydraulic conditions and erosion. Spur dikes are built using permanent materials like masonry, concrete, or earth and stone; semi-permanent materials like steel or wood sheet piling, gabions, or timber fence; or temporary materials like weighted brushwood fascines^15^, and regular maintenance is required to ensure their effectiveness. Despite its prevention, spur dikes substantially impact the river flow behaviour around the structures, leading to substantial changes in sediment movement, scour formation, and sediment accumulation near the structure.

Flow characteristics play a crucial role in shaping scour formations. Different shapes of spur dikes can lead to diverse scour patterns. Studies have shown that scour shape characteristics have implications for measuring and addressing scour issues^16, 17^. A restriction to the flow through the spur dike creates an intense and complex vortex system, inducing scour at the tip of the spur dike, which affects the strength of the structure^18^. According to^19^, the primary vortex impacts the bed near the spur dike, leading to erosion of the bed material and subsequent downstream transportation by the main flow. This results in scouring at the tip of the spur dike, and the scoured material is deposited elsewhere downstream section^20, 21^. Excessive separation of sediment particles from the base of the spur dike is defined as local scour. It significantly contributes to the weakening of the spur dike, the leading cause of the collapse. So, considering the expected maximum scour depth at near spur dike is important in avoiding such failure.

The problem becomes even more complex when seepage in the downward direction is involved. In this seepage process, the free water available under the ground moves inside the soil under gravity and occurs when there is a difference in hydraulic head. Downward seepage can further complicate the problem by exerting an additional hydrodynamic force on the sediment particles and changing the hydrodynamic parameters of the channel. Previous researchers found that downward seepage plays a vital role in modifying the channel’s morphology^22, 23^. Researchers suggested that permeable channels allow considerable water loss through the channel boundary, reducing the conveyance efficiency^24–26^. Alluvial channel seepage losses are estimated to be between 15 and 25% of total input^27^. According to^28^ research, half of the seepage flow is lost through the streambed. They suggest that a significant portion of the water that seeps into the ground and flows towards a stream is lost through the streambed. The study by^23^ examined the effect of downward seepage on the mean streamwise velocity in curved channels. They observed that the presence of downward seepage caused an increase in the mean streamwise velocity at the centre of the channel. Specifically, the mean streamwise velocity for trapezoidal channels increased by an average of 20%, while for rectangular channels, it increased by 26%. In addition, the downward movement of seepage enhances the bed particles’ transport rate and changes the channel’s hydrodynamic parameters. This has been demonstrated in several studies^29–36^. Thus, studying and analysing seepage processes in alluvial channels and correctly assessing them is important for properly designing structures. It is crucial to consider seepage’s effects when assessing riverbanks’ stability and developing erosion control strategies.

However, based on the currently available research, seepage has not been considered when assessing the channel morphology and turbulence near spur dike fields of alluvial channels. Thus, the present work reported the effect of downward seepage to analyse its effect on the morphology of sediment beds and the flow behaviour around the T-shaped spur dike field. In addition, this study examined the maximum scour depth, temporal scour depth, and the maximum scour depth’s location with and without downward seepage.

## Experimental setup and method

### Experimental details

**Figure 1:**
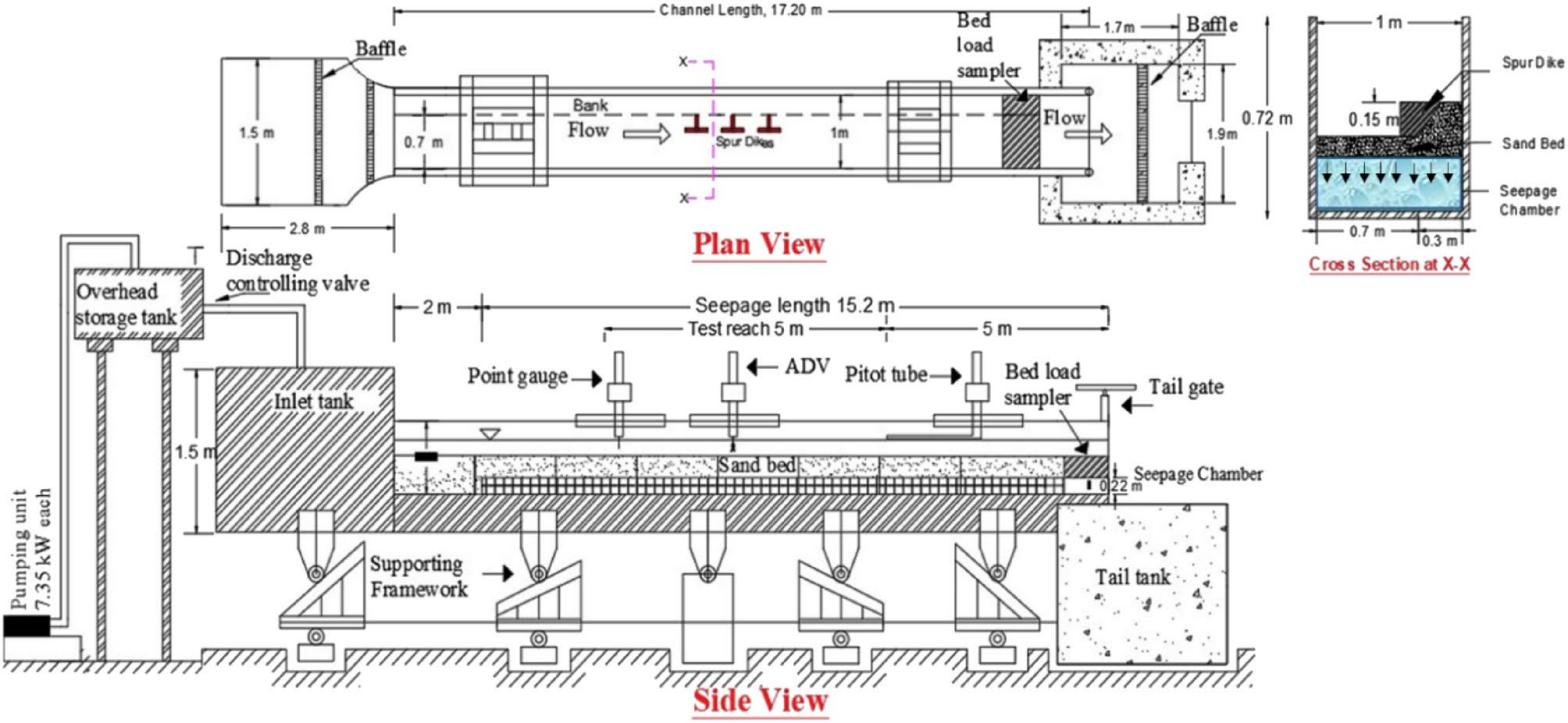
Experimental setup with spur dikes.

The experimental flume established in the Fluvial Dynamics lab under the Civil Engineering Department at the Indian Institute of Technology, Guwahati, was a critical tool in conducting various experiments related to fluvial dynamics. This facility was designed specifically to study water behaviour in river systems, and it proved to be an incredibly useful resource for researchers. The experimental flume was constructed to precise specifications to ensure the most accurate and reliable data collection possible. The flume was 17.2 m long, providing ample space for researchers to conduct various experiments. It was also 1 m wide, allowing for a wide range of flow rates and water depths to be studied. The flume was 0.72 m deep, with a 0.22 m-deep seepage chamber below the sediment bed. This seepage chamber allowed for measuring the suction (downward seepage) into the sediment bed. Figure 1 shows the experimental setup with an overhead water tank with three water pumps upstream of the flume. A lower-head tank is present downstream of the flume with a rectangular notch to measure the water head. The bed is prepared with sand size d_50_ = 1.1 mm. The channel is provided with a one-sided bank with a slope of 45 degrees, and the other side has a glass wall. The arrangement of the spur dikes included three consecutive spur dikes forming a spur dike zone. The T-shaped spur dikes extended from the bank, partially submerged, and aligned perpendicularly. Each spur dike had a projected length of 12.6 cm, which is equivalent to the length of the wing of the spur dike (L/L′ = 1). The total height of the spur dikes was 28 cm, with 15 cm exposed above the sediment bed and 13 cm submerged within it. The spur dikes were made with wood and located 8.5 m upstream from the channel’s end.

After establishing the experimental conditions, water is allowed in the channel gradually to avoid initial flow and bed disruption disturbance. The experiment was performed for two different discharge condition, and for every discharge, there were No-seepage; 5% and 10% seepage were applied. Almost similar flow depths were maintained for both these discharges. Two digital electromagnetic flow meters at the channel’s downstream end monitor the quantity of seepage applied. These flow meters are attached through the seepage chamber with the same diameter circular pipes. Each experimental run lasted 24 h or until the equilibrium condition was achieved. Achieving the equilibrium state at the scour hole near the spur dike tip was complex. Researchers defined this complexity in various ways^37^. State that an equilibrium state is to be considered when scour depth does not cross 1 mm after 3 h.

Further^38^, proposed that the experimental run be stopped if scour depth changed by less than 1 mm in 1 h. The present study observed that scour depth initially increased significantly during 0–6 h; it grew moderately 6–18 h and minorly over 18–24 h. The change in scour depth during 22–24 h did not exceed 1 mm. The experiments were conducted till the equilibrium condition was reached. The flow was stopped after an equilibrium stage had been reached, and the bed was allowed adequate time to enable the water to drain.

### Data collection and processing

**Figure 2:**
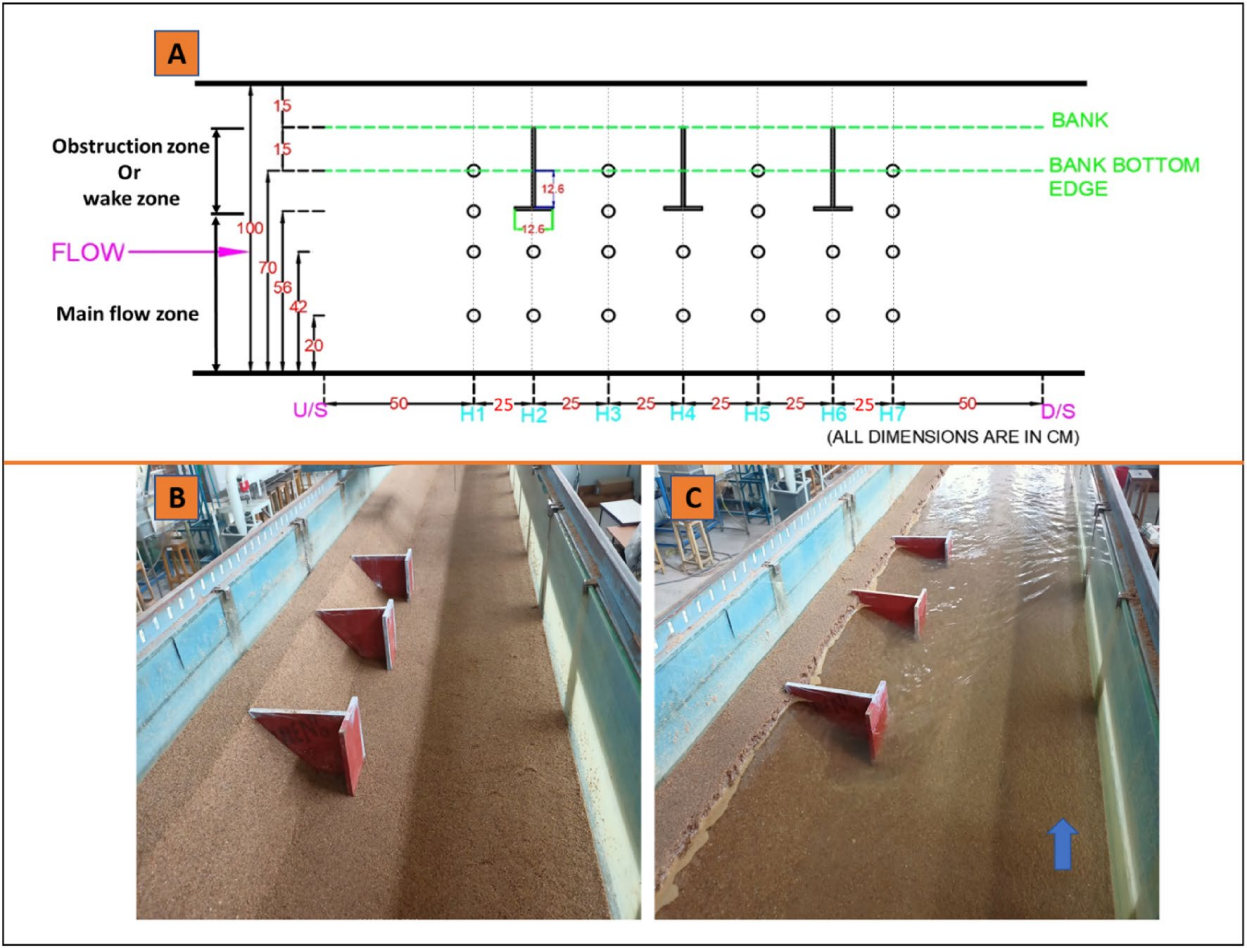
(**A**) Shows the velocity measuring points at different locations of spur dike zones, (**B**) Snapshots of experiments without flow from the upstream point of view, (**C**) Snapshots of experiments with flow from the upstream point of view.

In the present study, instantaneous velocity was measured at 22 locations, as shown in Fig. 2 (A), and for measuring the instantaneous flow velocities around spur dikes using a four-beam down-looking acoustic Doppler velocimeter, or ADV, made by a company called Nortek. The three-dimensional velocities are measured into three components: u, v, and w, which represent the streamwise (X), transverse (Y), and vertical (Z) directions, respectively. The data collection process involved taking measurements for 2 min at each depth point, with a sampling frequency of 200 Hz. This means the ADV recorded 200 measurements per second at each depth point for 2 min. Once the velocity data was collected, it was filtered using a data filtration method called^39^ acceleration threshold. This method involves identifying and removing high-frequency noise and motion-related artefacts from the velocity data by applying an acceleration threshold. This step aims to ensure that the filtered data reflects the true velocity of the fluid at the depth point. After applying the filtration method, the velocity power spectra of the filtered data were analysed to check if they matched the − 5/3 rule of Kolmogorov^40^. The − 5/3 rule of Kolmogorov is a fundamental scaling law in fluid dynamics that describes the energy spectrum of turbulent flows. Once the data has been filtered, it can be used for further analysis of turbulent characteristics. The analysis of turbulent characteristics involves the examination of various properties related to turbulence, such as mean velocities, Reynolds shear stress, turbulent kinetic energy, and turbulent intensities. These parameters help understand the nature and behaviour of turbulence in the system under investigation. The accuracy of the Acoustic Doppler Velocimeter (ADV) was evaluated by measuring the standard deviation and uncertainty of average mean velocities and Reynolds stresses (Table 1). This was achieved by collecting 15 samples of velocity data (at z/h = 0.11) in the test section. Accordingly, this instrument is accurate, as the flow characteristics have very low uncertainty.

**Table 1:** Uncertainty analysis of data recorded by ADV.

	$\overline{u}$ (m/s)	$\overline{v}$ (m/s)	${\overline{\text{w}}}$ (m/s)	$\left( {\overline{{{\text{u}}^{\prime } {\text{u}}^{\prime } }} } \right)^{0.5}$ (m/s)	$\left( {\overline{{{\text{v}}^{\prime } {\text{v}}^{\prime } }} } \right)^{0.5}$ (m/s)	$\left( {\overline{{{\text{w}}^{\prime } {\text{w}}^{\prime } }} } \right)^{0.5}$ (m/s)
Standard deviation	$4.6 \times {10}^{-3}$	$3.9 \times {10}^{-5}$	$3.1 \times {10}^{-5}$	$4.24 \times {10}^{-4}$	$3.41\times {10}^{-4}$	$1.9 \times {10}^{-5}$
Uncertainty (%)	0.215	0.156	0.140	0.024	0.039	0.131

Figure 2A shows the 22 locations where the velocity was measured in the spur dike field. The measurement points were established near the spur dike field and its surroundings to study the flow behaviour. For that, two zones are specified where the measurements were taken: obstruction zone (plane Y = 56–85) or wake zone and main flow zone (plane Y = 0–55). The Velocity measurement was taken in Y-plane as Y = 0.70 m, Y = 0.56 m, Y = 0.42 m, and Y = 0.20 m and in X = 0, 0.25, 0.50, 0.75, 1, 1.25, and 1.50 m as shown in Fig. 2A. Where X = 0 represent the section (H1) upstream of the spur dike-1, X = 0.25 section (H2) at spur dike-1 and X = 0.50 section (H3) downstream of spur dike-1, X = 0.75 section (H4) at spur dike-2, X = 1 section (H5) at downstream of spur dike-2, X = 1.25 section (H6) at spur-3 and X = 1.50 section (H7) at downstream of the spur dike-3. At each location’s depth-wise, around 18–22 readings were collected.

Figure 2B,C shows the experimental run before and during the flow condition. Experiments were carried out with two discharges, Q_1_ = 0.023 m^3^/s and Q_2_ = 0.030 m^3^/s, to investigate the influence of seepage on bed morphology. A digital point gauge was utilised to determine the extent of bed deformation, which had a least count of 0.01 mm. This instrument enabled the measurement of the elevation of the channel bed with respect to a reference datum. The point gauge was mounted on a mobile trolley, which allowed it to be easily transported to any desired location to take measurements. To construct the morphology, elevation data were collected every 5 cm in the longitudinal direction up to a length of 1.5 m and every 2–5 cm (varying based on undulations) in the transverse direction up to a width of 1 m. The measurements were obtained to cover the entire spur dike field. Table 2 shows the experimental flow parameter details.

**Table 2:** Flow parameter details.

Inflow discharge Q (m^3^/s)	Seepage case	Mean velocity, V (m/s)	Seepage discharge Q_s_ (m^3^/s)	Froude number, Fr	Reynolds number, Re
Q_1_ = 0.023	No-seepage	0.254	–	0.23	24,155
	5% S	0.262	0.0012	0.24	24,916
	10% S	0.311	0.0023	0.28	29,576
Q_2_ = 0.030	No-seepage	0.303	–	0.27	29,694
	5% S	0.316	0.0015	0.28	30,968
	10% S	0.334	0.0030	0.3	32,732

## Results and discussions

This investigation aimed to analyse the impact of downward seepage on the hydro-morpho dynamics process around the spur dikes. The investigation focused on the impact of three T-shape spur dikes with varying percentages of seepage, i.e., No-seepage, 5%, and 10% seepage. This section also presents the variation of bed morphology, longitudinal profile, and the temporal evolution of scour depth for two different discharge rates, specifically Q_1_ = 0.023 m^3^/s and Q_2_ = 0.030 m^3^/s, both with and without downward seepage. In addition to these observations, the turbulent flow characteristics such as streamwise velocity, Reynolds shear stress, and turbulent kinetic energy were examined in the zone of spur dikes under seepage and non-seepage conditions.

To understand the impact of downward seepage, the paper investigated turbulent flow characteristics in two horizontal planes near the water surface z/h = 0.55 and the channel bed z/h = 0.1. The flow properties near the water surface were important for modeling hydrodynamic processes, while the channel bed was susceptible to erosion and channel migration due to seepage. The study aimed to address these issues and gain insight into how seepage affects turbulent flow patterns in the channel.

### Morphological changes with T-shaped spur dikes

**Figure 3:**
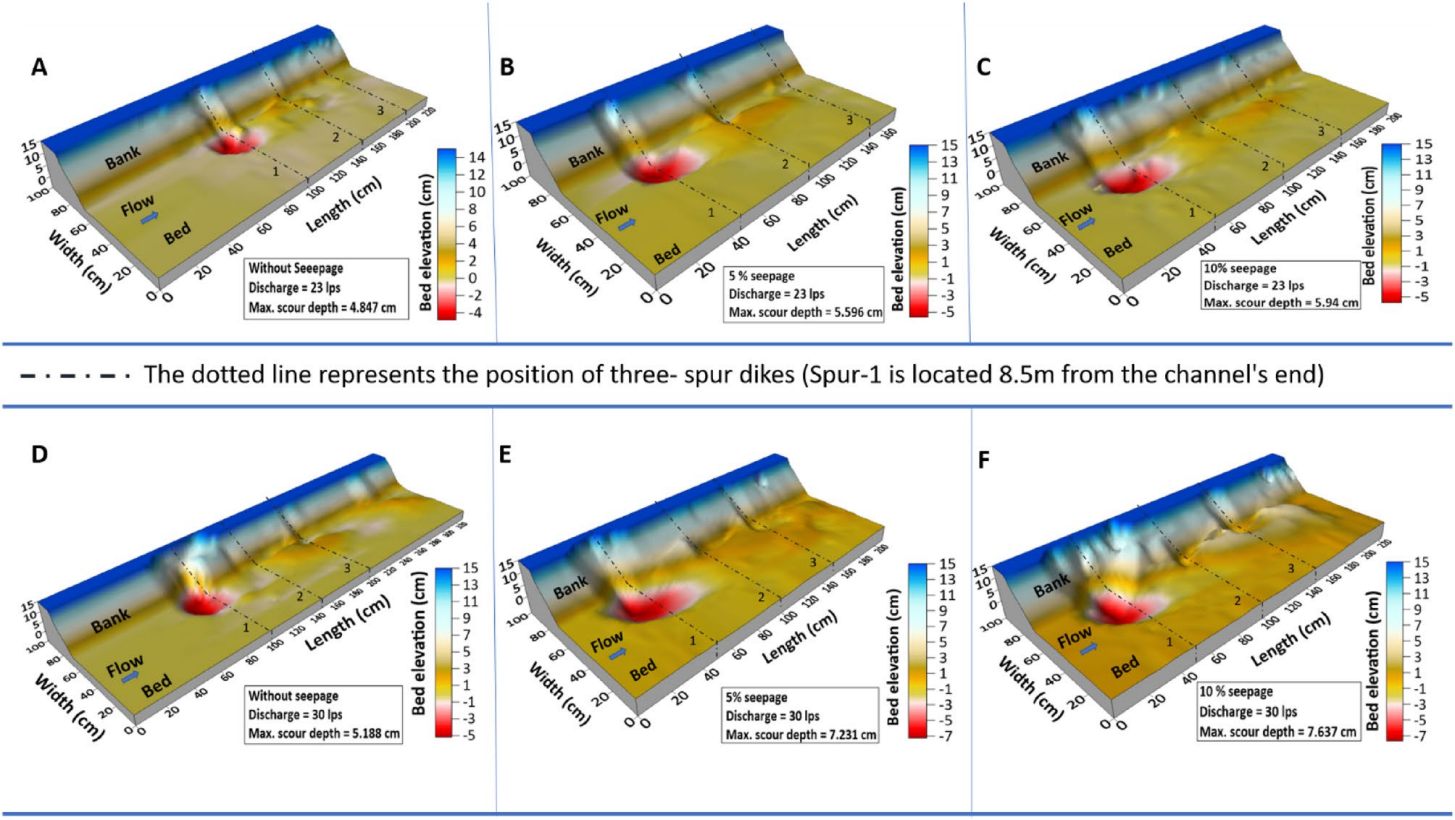
Shows the 3d surface plots around the spur dike field for two distinct flow discharges, i.e. Q_1_ = 0.023 m^3^/s (**A**, **B**, **C**) and Q_2_ = 0.030 m^3^/s (**D**, **E**, **F**) and for no-seepage, 5%, 10% seepage.

Figure 3 shows the bed morphology change around the test section’s spur dike field. According to the findings, seepage considerably impacts the whole geometry of the alluvial channel. The application of seepage enhances the movement of sediment particles, and when the seepage is increased, the detachment of particles increases, creating a deep scour hole. The maximum local scour was identified near the tip of spur-1’s wing, with minor scour depth reported at the place of spur dike 2 and 3. In all cases, scour material detached from the first spur dike was deposited near spur dike-3. As a result, a dune-like structure forms near the spur dike-3. The scour depth is also extended along the longitudinal direction with the increment of the seepage rate.

The measurements were taken at two different discharge rates, Q_1_ = 0.023 m^3^/s and Q_2_ = 0.030 m^3^/s, under three conditions: no seepage, 5% seepage, and 10% seepage. In Q_1_, the maximum scour depth was observed to be 4.84 cm, 5.59 cm, and 5.94 cm for no seepage, 5% seepage, and 10% seepage, respectively. This indicates that the maximum scour depth also increased as the seepage increased. Specifically, with the application of 5% seepage, the maximum scour depth was raised by 15.5%, and with 10% seepage, it increased by about 22.7% compared to the no-seepage condition.

Similarly, in Q_2_, the maximum scour depth was measured to be 5.18 cm, 7.23 cm, and 7.62 cm for no seepage, 5% seepage, and 10% seepage, respectively. Here, the maximum scour depth was found to increase significantly with the application of seepage. With 5% seepage, the maximum scour depth grew by 39.5%, and with 10% seepage, it increased by 47%, compared to the no-seepage condition. Overall, the results suggest that seepage significantly impacts the maximum scour depth in an alluvial channel, with higher seepage rates leading to greater scour depths. The variation in channel morphology and bed distorted more with seepage. Additionally, the effect of seepage appears to be more pronounced at higher discharge rates.

### Variations in bed morphology

**Figure 4:**
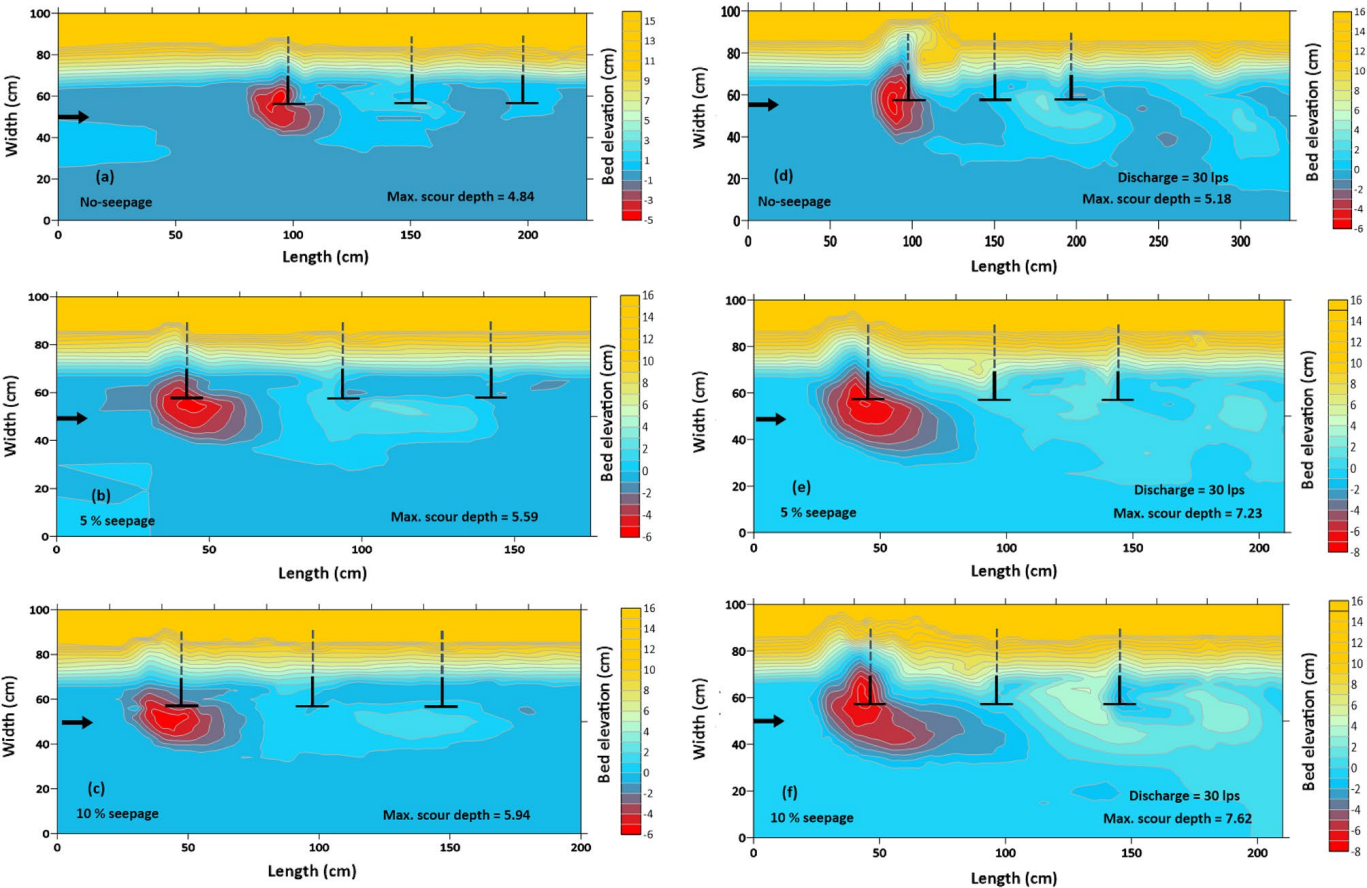
(**a**, **b**, **c**) Contour plots of the morphological changes for discharge Q = 0.023 m^3^/s with no seepage and 5% and 10% seepage. (**d**, **e**, **f**) Contour plots of the morphological changes for discharge Q = 0.030 m^3^/s with no seepage and 5% and 10% seepage.

Figures 4 show the contour plots of the experimental run’s test section for discharges Q_1_ = 0.023 m^3^/s and Q_2_ = 0.03 m^3^/s under no-seepage, 5% seepage, and 10% seepage circumstances. The position of the spur dikes is located in the representation of contour plots. The three spur dikes were erected in sequence, projecting outward from the bottom edge of the bank and spaced 50 cm apart. As the streamflow approached the spur dike, it diverted through the edge of the spur dike-1. Thus, the spur dike facing the streamwise velocity attained more scour depth than the others in this sequence. The contour plots reveal that the maximum scour depth occurs near the first spur dike. Red-coloured projection of contours shows the scour hole and their expansion originates from the tip of the spur dike-1. The diameter of the scour hole expanded along the spur dike field. The larger and deeper diameter scour hole was observed with the application of seepage. The scour material deposited downstream of the test section resulted from flow strength reduction, forming a dune-like structure between the second and third spur dikes. The measurements were taken to the extent that the bed morphology was altered over the channel length.

### Longitudinal profile of the bed deformation

**Figure 5:**
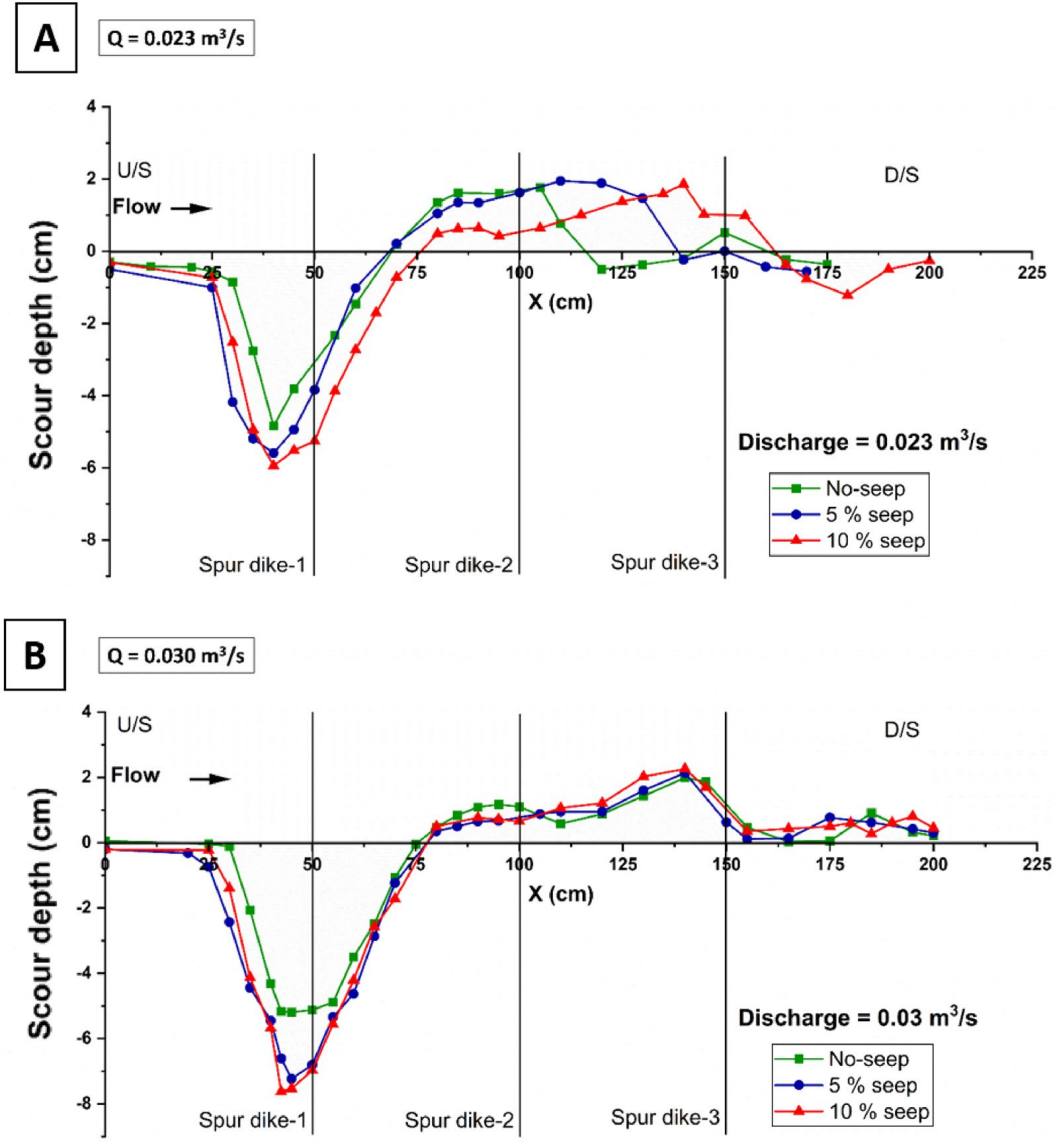
Scour depth variation along the length of the test section with no seepage, 5% seepage, and 10% seepage with a different flow rate of Q_1_ = 0.023 m^3^/s (**A**) and Q_2_ = 0.030 m^3^/s (**B**).

The longitudinal profiles of Fig. 5 show the bed elevation variation along the flow direction of two different discharges: Q_1_ = 0.023 m^3^/s (A) and Q_2_ = 0.030 m^3^/s (B) with seepage and without seepage conditions. Elevation points were observed along the line, where the maximum scour depth was achieved. Figures show that the maximum scour depth is achieved near spur-1, and the bed elevation rises as the percentage of seepage increases compared to the no-seepage case. The diameter of the scour hole also expanded with seepage. The length of deposition also increases with seepage. The maximum deposition observed between the second and third spur dike and the deposition height increases as seepage is applied.

### Temporal development of scour depth

**Figure 6:**
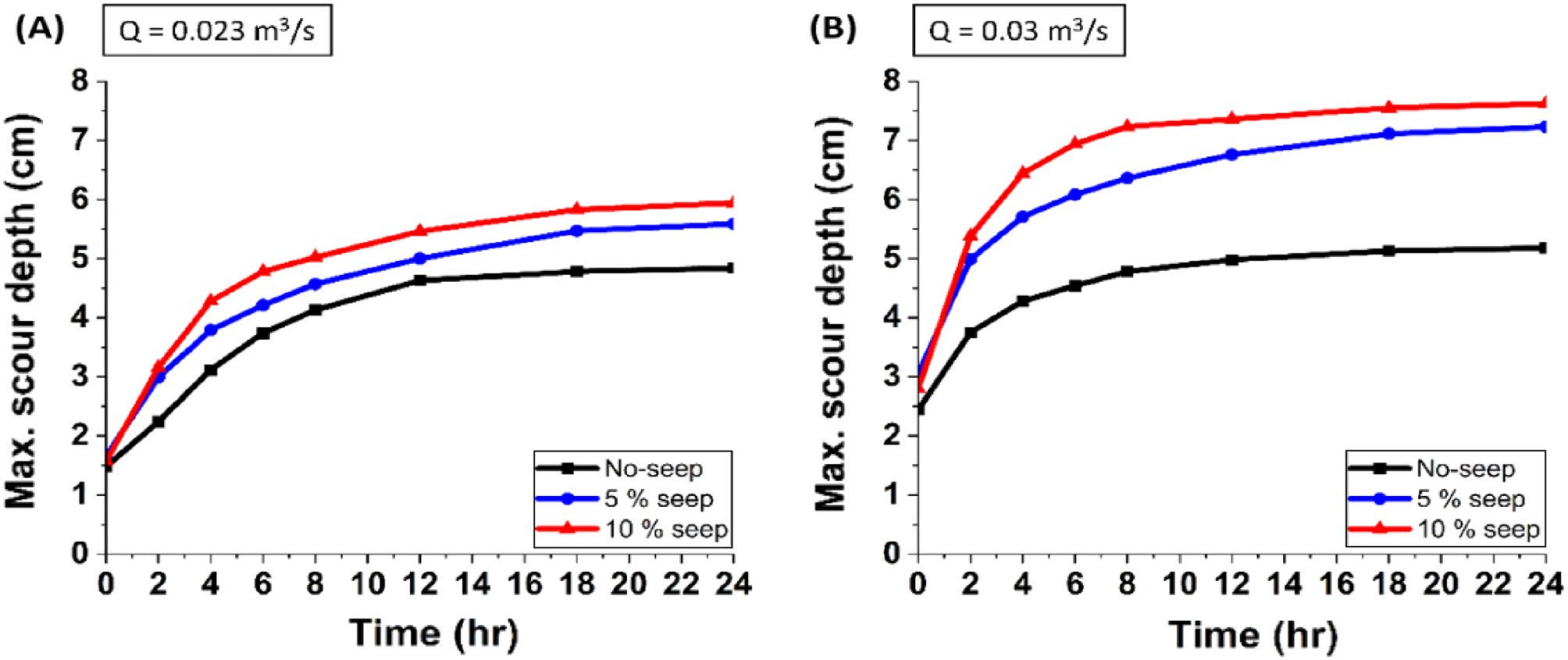
Variation of temporal scour depth at the tip of spur dike-1 with no seepage, 5% seepage, and 10% seepage for each discharge of Q_1_ = 0.023 m^3^/s (**A**) and Q_2_ = 0.030 m^3^/s (**B**).

Figure 6 shows the variation of scour depth with time at different seepage percentages (0%, 5%, and 10%) and two discharge rates (Q_1_ = 0.023 m^3^/s and Q_2_ = 0.030 m^3^/s). The results show that the maximum scour depth occurs at the tip of the spur dike-1, and the change in scour depth is rapid in the initial six hours, after which it gradually decreases over time and attains equilibrium after 18 h. This pattern also follows the same trend for other discharges. The variation in scour depth is greater with seepage and increases with seepage percentage.

**Figure 7:**
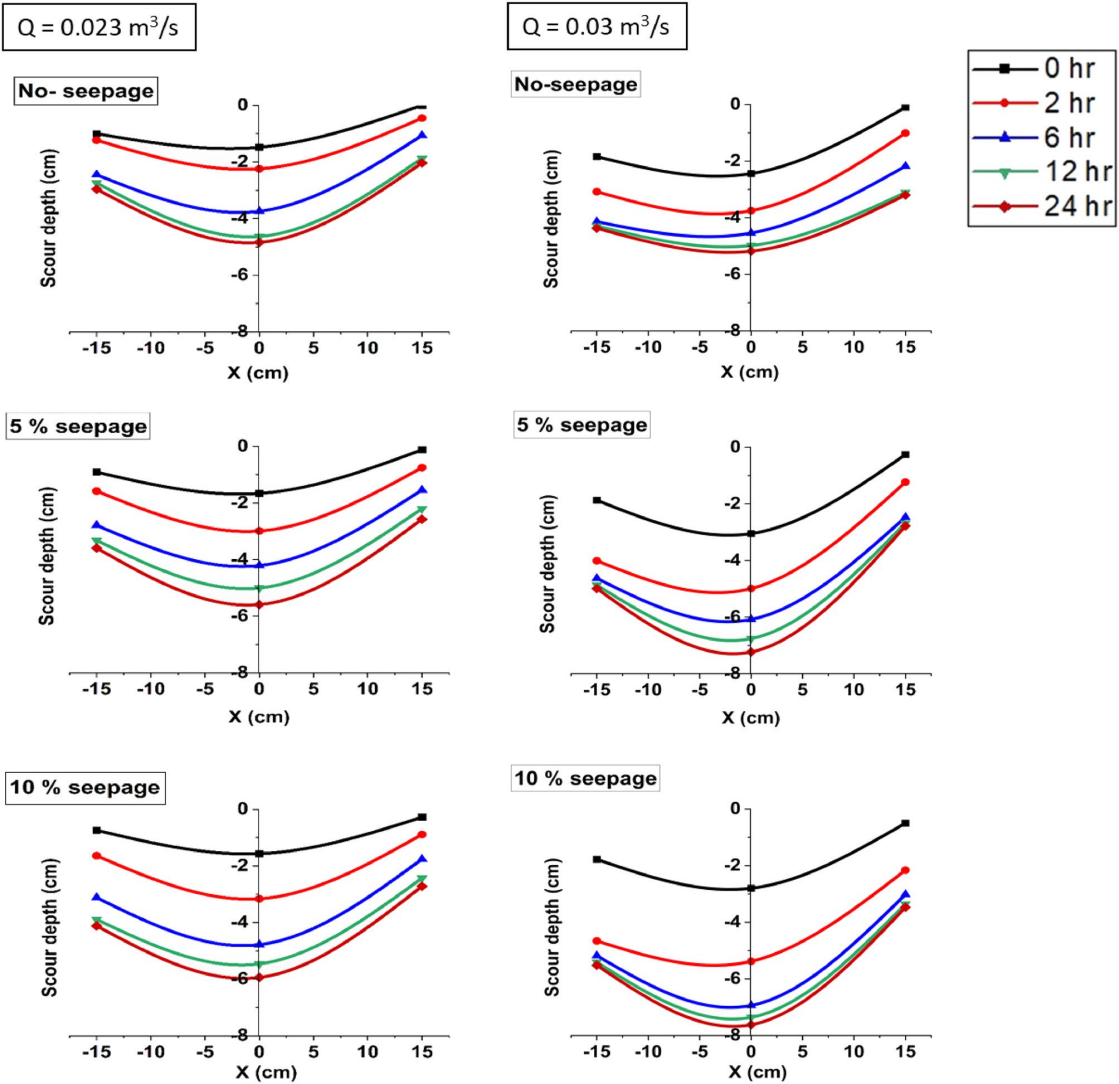
Development of scour depth with time near spur dike-1 for different discharges and with and without seepage (bed level considered zero level).

Figure 7, on the other hand, illustrates the evolution of scour depth with time at the tip of the spur dike along the flow direction for a specific discharge rate and given seepage condition. The elevation data were collected at three locations: at the edge of the spur dike (where maximum scour depth was found), 15 cm upstream, and 15 cm downstream to the edge of the spur dike. Applying flow rate and seepage resulted in scour depth’s rapid development.

Overall, the study emphasises the significance of considering the effects of seepage on scour depth at spur dikes. The findings suggest that the scour depth variation is significant and increases with seepage percentage.

The presence of spur dikes or groynes significantly impacts river flow patterns, modifying the shape and structure of the channel by facilitating sediment movement and inducing general and local scour while promoting deposition around the spur dike field. As the flow encounters the spur dike field, an obstruction is created, resulting in the formation of a bow wave at the water’s surface. This wave generates a downward flow towards the bottom of the channel. Additionally, a horseshoe vortex forms at the boundary of the obstruction, while a wake vortex system develops behind the spur dike.

These vortices play a crucial role in dislodging sediment particles from the structure’s base and propelling them through the increased lateral flow that passes through the obstruction. Consequently, the sediment becomes loose and is carried downstream by the river’s main flow. Eventually, this sediment accumulates and settles in downstream sections of the river where the velocity of the water is relatively low^20, 21^.

## Flow characteristics

### Velocity

Two zones are considered in the flow field: the main flow zone and the obstruction zone. The main flow zone (y = 0–56) is where the approaching flow converges through the spur dikes and flowing, while the obstruction zone (y = 56–85) is where the spur dikes restrict flow. The velocity is measured at seven cross sections (H1, H2, H3, H4, H5, H6, and H7), with 3–4 points established in each section, as shown in Fig. 2A.

**Figure 8:**
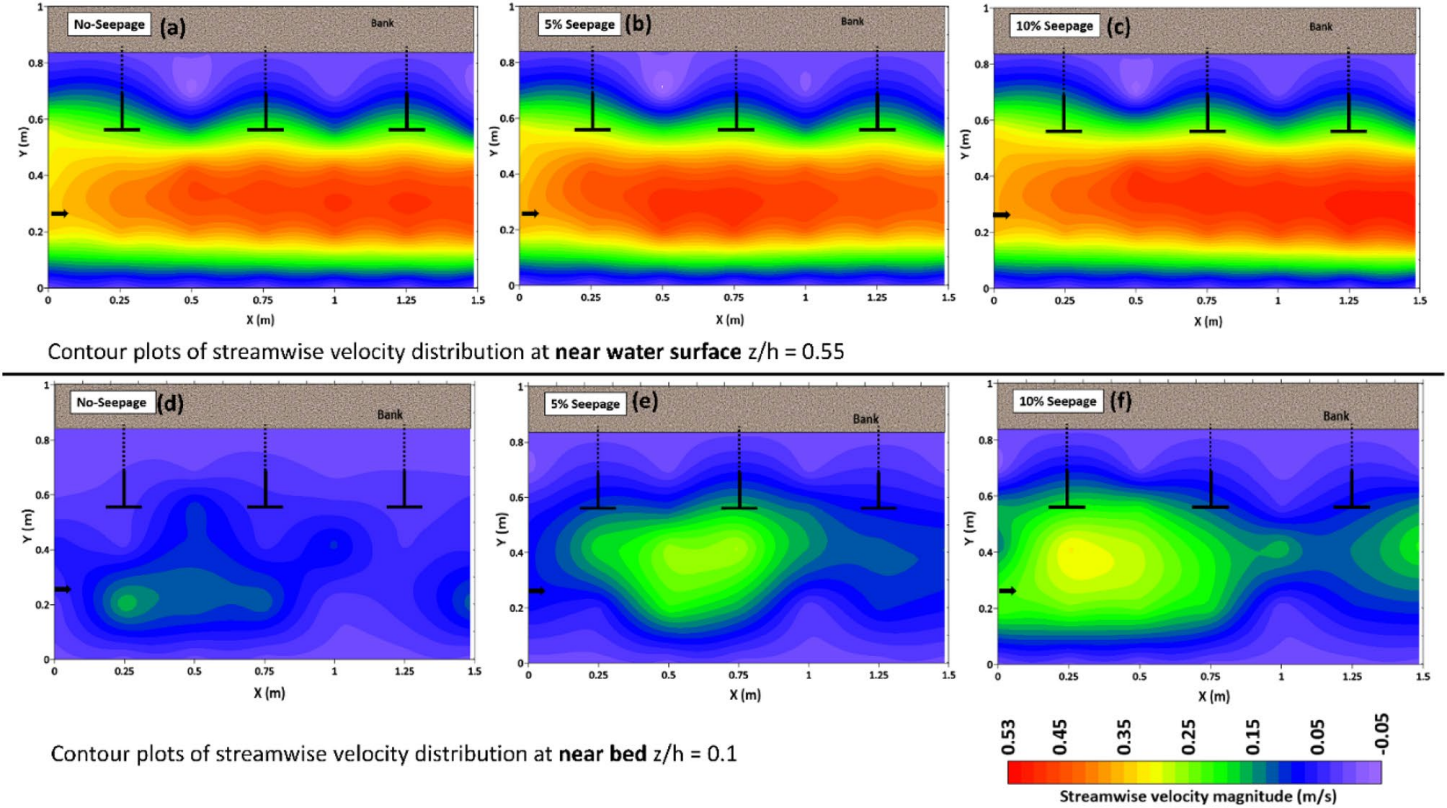
Shows the contour plots of the streamwise (x-direction) velocity distribution of the spur dike field near the water surface at z/h = 0.55 (**a**, **b**, **c**) and the bed of the channel at z/h = 0.1 (**d**, **e**, **f**).

Figure 8a–c shows the streamwise velocity distribution contour plots near the water surface z/h = 0.55. The figure shows that as the flow approaches spur dike zones, flow is diverted and constricted through the edge of the spur dike’s wing facing the flow. Thus, the velocity of flow increases in the main flow zone. This increased velocity is distributed and expanding in the longitudinal direction from the edge of spur dike-1. The maximum velocity magnitude is observed near spur dike-1, which is reduced after the entire field of spur dike. The flow strength decreases as it diverges downstream of the spur dike zones. The least magnitude of velocity or negative velocity observed in the obstruction zone. In addition, the velocity magnitude is higher in the main flow zone with downward seepage compared to the no seepage condition.

Figure 8d–f represents the streamwise velocity distribution contour plots near the channel bed at z/h = 0.1. The velocity magnitude near the bed is very low in the absence of seepage due to bed resistance in the channel boundary. However, it is evident from the figure that the velocity magnitude near the bed of the channel is significant in the main flow zone with the effect of seepage, which is responsible for enhancing the sediment transport rate. With downward seepage, the flow distribution has shifted downward at all measurement locations. Seepage increases the velocity magnitude near the channel bed and decreases particle stability, with the highest magnitude observed near spur dike-1 with 10% seepage. The velocity variation near the boundary of the channel is insignificant in the obstruction zone. The downward seepage is crucial in modifying the flow pattern near the channel bed and influencing the morphology of the alluvial channel.

**Figure 9:**
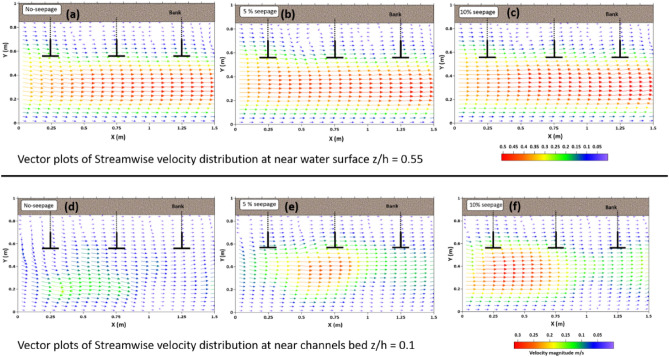
Vector plot showing velocity distribution (streamwise and transverse) at water Surface z/h = 0.55 (**a**, **b**, **c**) and near bed z/h = 0.01 (**d**, **e**, **f**) of discharge Q = 0.03 m^3^/s with No-seepage, 5% seepage and 10% seepage.

Figure 9 displays velocity vector plots for water flow near the surface z/h = 0.55 (a, b, c) and bed z/h = 0.1 (d, e, f) of a channel with and without seepage. The plots demonstrate that as the flow encounters a spur dike, a stagnation zone forms, obstructing the flow and creating a bow wave at the water’s surface. Meanwhile, a horseshoe vortex emerges at the base of the spur dike, and a wake vortex system develops behind the spur dike. The vortices cause the sediment particles to become unsteady and detach from the structure’s base, which is then transported downstream with the main flow. Very low-velocity magnitudes of positive and negative values were observed in the wake zone between the spur dikes. This confirms cross-stream circulation and secondary current generation inside the loop. At downstream (behind the spur dike) the spur dike field, a negative velocity is observed due to the backwater effect. The velocity distribution at the channel bed shows that the streamwise velocity magnitude increases near the channel’s boundary due to downward seepage. These observations align with previous research conducted by^32–34^.

The study showed that the most variation in the flow field is found near the spur dikes, at plane Y = 42 m, such that this paper compares the flow properties at this plane of seven different sections. At the near water surface z/h = 0.55, the highest value of streamwise velocity magnitude was observed at section H3-42 for all cases, including no seepage, 5% seepage, and 10% seepage. The magnitude percentage increase was 4% and 7.9% for 5% and 10% seepage, respectively, compared to no seepage.

However, near the bed z/h = 0.1, where seepage has a more significant effect, the maximum value of streamwise velocity magnitude was observed at section H4-42 for no-seepage and 5% seepage, and section H2-42 for 10% seepage. The increment in magnitude variation found for 5% and 10% seepage was 1.85 times and 2.24 times, respectively, compared to the case with no seepage.

### Reynolds shear stress

**Figure 10:**
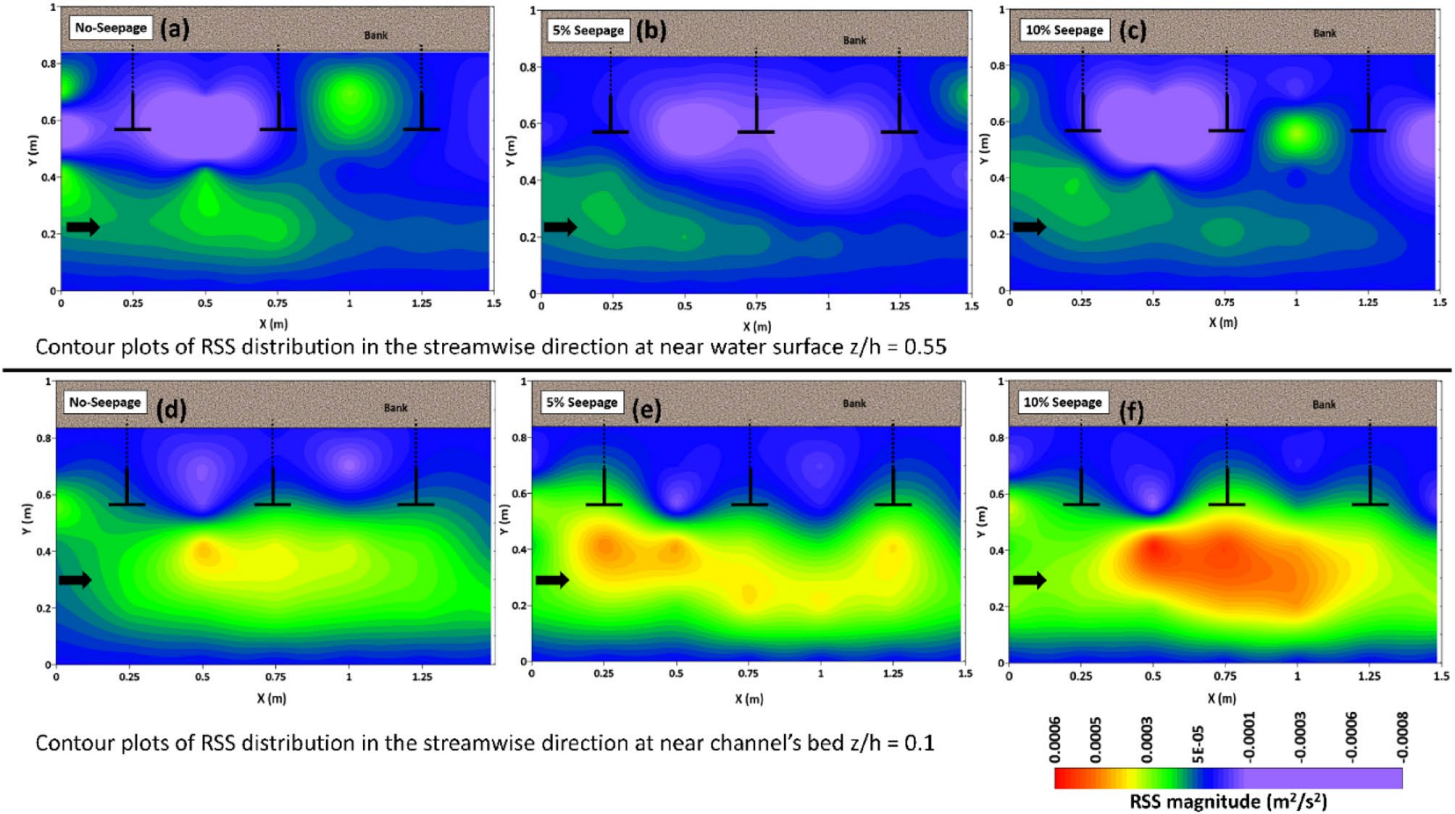
Displays the contour plots of the Reynolds shear stress ($- \overline{{u^{\prime}w^{\prime}}}$) distribution of the horizontal plane for no seepage, 5%, and 10% seepage near the water surface at z/h = 0.55 (**a**, **b**, **c**) and the channels bed at z/h = 0.1 (**d**, **e**, **f**).

The results of this section provide an insight into the Reynolds shear stress (RSS) distribution in the streamwise direction $\tau_{uw} = - \rho \overline{{u^{\prime}w^{\prime}}}$, which acts on the X–Y plane and is parallel to the flow direction. Here, u′ and w′ represent the turbulent fluctuation components of the instantaneous velocities in the streamwise and downward directions. Figure 10 shows the contour plots of the RSS distribution at different horizontal planes, i.e., near the water surface z/h = 0.55 (a, b, c) and the bed z/h = 0.1 (d, e, f) are crucial in understanding the flow characteristics with and without seepage.

The experiments show that the maximum stress is found in the main flow zone near the spur dike when the RSS distribution is measured near the bed (z/h = 0.1). Seepage increases turbulence and mixing near the boundary, which improves momentum and sediment particle transfer. The result supports previous research that the Reynolds stress has a characteristic triangular distribution near the channel boundary. The maximum stress occurs at the boundary and decreases with increasing distance from the boundary^41^.

The presence of seepage amplifies the Reynolds stress near the bed, leading to an increase in bedload transport in the main flow zone. The results also highlight the discontinuity in the RSS distribution near the water surface in the spur dike field. Most of the region in the obstruction zone is dominated by negative stresses, indicating the formation of vortices inside the wake zone. The flow momentum increases due to flow constriction as it reaches the spur dike zone, resulting in stronger intermixing of the turbulent flow. The fluctuation of the RSS is reduced as the flow passes the spur dike field, and the strength of the flow decreases, due to which deposition occurs at the end of the spur field.

In the obstruction zone of the spur dike, the RSS is highly fluctuating due to the formation of wakes. The magnitude of the RSS in the streamwise direction shows positive and negative low values, which confirm cross-stream circulation. In addition, the dynamics of horizontal turbulent mixing are stronger due to the large obstruction to the incoming flow.

Regarding the maximum magnitude of RSS distribution, it was observed at section H1-42 for no-seepage, 5%, and 10% seepage at the near water surface. The magnitude decreased by 0.5 and 0.4 times for 5% and 10% seepage, respectively, compared to the case with no seepage. However, near the bed, the maximum magnitude of RSS distribution was observed in sections H3-42 for all the cases. The magnitude variation found for 5% and 10% seepage was 15% and 0.5 times increment, respectively, compared to no seepage. The magnitude of the RSS decreases near the water surface and increases towards the channel bed to overcome bed resistance and keep the sediment particles in motion.

### Turbulent kinetic energy

The turbulent kinetic energy (TKE) measures the energy contained within the turbulent motions of a fluid.

$${\text{It}}\,{\text{ is}}\,{\text{ given}}\,{\text{ by}}\,{\text{ the }}\,{\text{expression}}:TKE = \frac{1}{2}\left( {\overline{{u^{\prime}u^{\prime}}} + \overline{{v^{\prime}v^{\prime}}} + \overline{{w^{\prime}w^{\prime}}} } \right).$$

**Figure 11:**
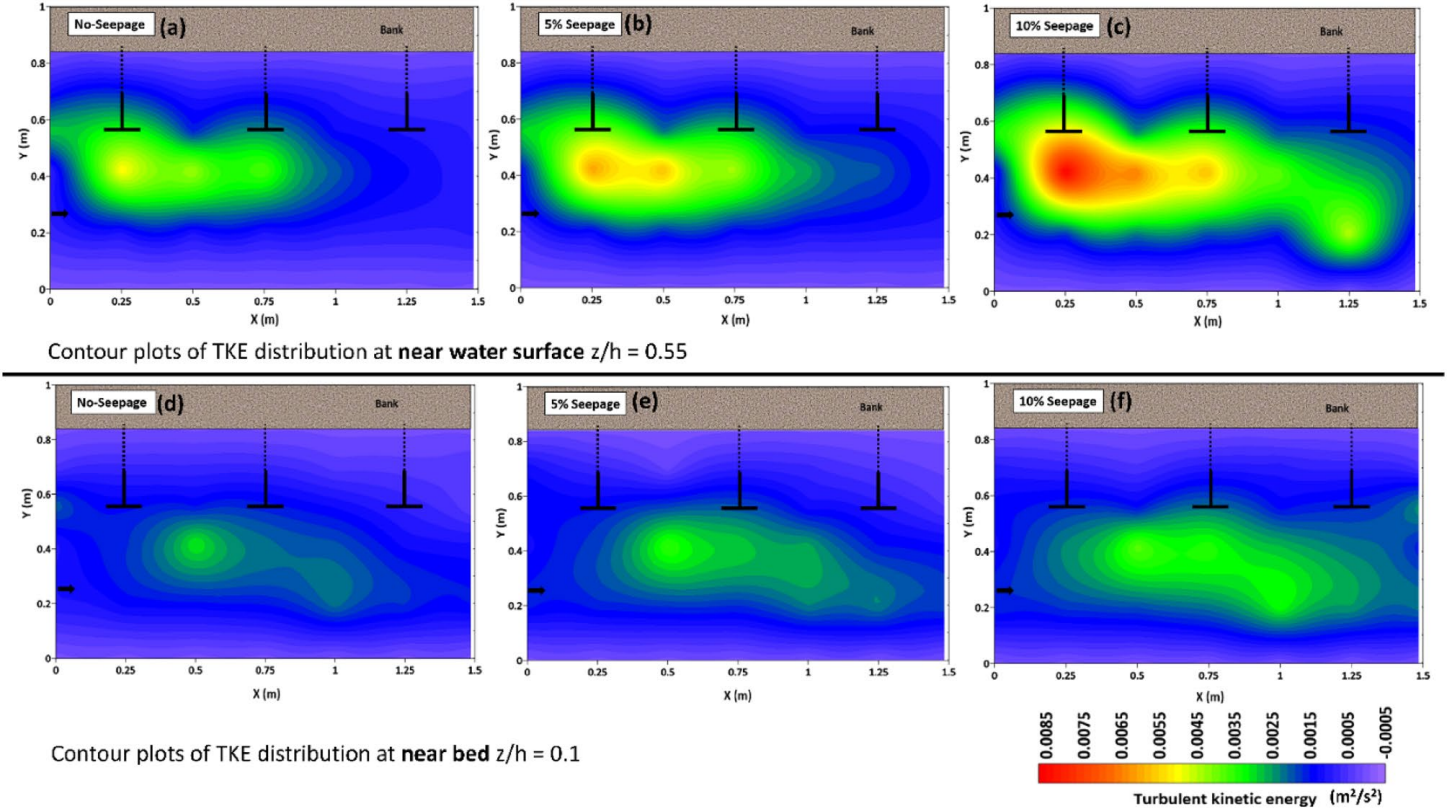
Shows the contour plots of the turbulent kinetic energy distribution of the spur dike field near the water surface at z/h = 0.55 (**a**, **b**, **c**) and the bed of the channel at z/h = 0.1 (**d**, **e**, **f**).

Here u′, v′, and w′ represent the fluctuations components of velocity in the x, y, and z directions, respectively. These components are deviations of the velocity from the mean flow and arise due to the turbulent fluctuations in the fluid. The overbar above each term in the expression represents time-averaging. Figure 11 shows the contour plots of the TKE distribution at different horizontal planes, i.e., near the water surface z/h = 0.55 (a, b, c) and the bed z/h = 0.1 (d, e, f). The TKE contours show that the maximum values of its magnitude are found near the bed, in the vicinity of the first spur dike and elongates towards the downstream. The magnitude of the TKE increases in the main flow zone where high velocity is observed; this supports the previous research study that found the highest TKE at high velocity in the separation zone^42^. However, TKE magnitude decreases in the obstruction or inside the wake zone. Very low or negative TKE magnitude values are observed in the obstruction zone. The most variation in the TKE is found at the tip of the first spur dike. The TKE magnitude increases with the application of seepage, and the maximum magnitude is observed with 10% seepage. The distribution of TKE magnitude is low in the horizontal plane at the water surface z/h = 0.55 and increases near the channel bed; it attains its maximum near bed z/h = 0.1.

Regarding the TKE distribution, at the near water surface, the maximum magnitude of TKE distribution was observed at section H2-42 for all cases, including no seepage, 5% seepage, and 10% seepage. The magnitude increased by 19.2% and 0.68 times for 5% and 10% seepage, respectively, compared to the case with no seepage. However, it was observed near the bed at section H3-42 for all no-seepage, 5%, and 10% seepage. The maximum variation of TKE magnitude for 5% and 10% seepage was 13% and 21%, respectively, compared to no seepage.

## Conclusion

This study sheds light on how flow behaves near a spur dike field in the presence and absence of downward seepage in two horizontal planes, namely those near the water’s surface (z/h = 0.55) and the channel bed (z/h = 0.1). The spur dike field is divided into the main flow zone and the obstruction zone or wake zone.

- The results demonstrate that downward seepage movement can cause significant modification throughout the channel geometry and the development of scour depth. At the base of the spur dike-1, the Horseshoe vortex forms, and a wake vortex system develops behind the spur dike. Thus, the tip of spur-1’s wing, which faces the flow directly, was found to have the greatest local scour. The vortices cause the sediment particles to become unsteady and detach from the base of the structure. In addition, the downward seepage enhances the movement of sediment particles, and when the seepage is increased, the detachment of particles increases, creating a deep scour hole. The diameter of the scour depth also increased longitudinally along with the seepage rate.
- The velocity distribution in a channel, noting that near the channel bed, the velocity is very low due to resistance from the channel boundary. However, the downward seepage has caused a shift in the flow distribution and increased the velocity near the bed in the main flow zone, enhancing the sediment transport rate. In contrast, very low-velocity magnitudes of positive and negative values were observed in the wake zone between the spur dikes. This confirms cross-stream circulation and secondary current generation inside the loop.
- The magnitude of the RSS increases near the channel boundary with the application of downward seepage. Seepage creates additional turbulence and mixing near the boundary, which enhances the transfer of momentum and sediment particles. In the obstruction zone of the spur dike, the Reynolds shear stress is highly fluctuating due to the formation of wakes. The component of RSS in the streamwise direction shows positive and negative low values, which confirm cross-stream circulation. The dynamics of horizontal turbulent mixing are stronger due to the large obstruction provided to the incoming flow.
- The TKE shows the maximum values of its magnitude near the bed, at around the first spur dike, and elongates towards the downstream. The magnitude of the TKE increases with seepage application, and the maximum magnitude is observed with 10% seepage.

While lab experiments offer valuable insights, it is crucial to acknowledge their limitations, particularly regarding their applicability to real-world situations. To address this, future studies should consider non-uniform sediment sizes, as this will lead to a better understanding of the complex interactions involved in downward seepage and scouring behaviour.

## Data Availability

The datasets used and/or analysed during the current study available from the corresponding author on reasonable request.
